# Usefulness of Quantitative Bone Single Photon Emission Computed Tomography/Computed Tomography for Evaluating Response to Neoadjuvant Chemotherapy in a Patient with Periosteal Osteosarcoma

**DOI:** 10.7759/cureus.3655

**Published:** 2018-11-28

**Authors:** Kazuhiro Kitajima, Hiroyuki Futani, Masayuki Fujiwara, Go Minakawa, Yuko Osugi, Tatsuya Tsuchitani, Koichiro Yamakado

**Affiliations:** 1 Radiology, Hyogo College of Medicine, Nishinomiya, JPN; 2 Orthopaedics, Hyogo College of Medicine, Nishinomiya, JPN; 3 Internal Medicine, Hyogo College of Medicine, Nishinomiya, JPN

**Keywords:** bone scintigraphy, quantitative spect/ct, periosteal osteosarcoma, standardized uptake value (suv)

## Abstract

We report here a case of periosteal sarcoma in a 10-year-old female, along with quantitative values obtained with bone single photon emission computed tomography/computed tomography (SPECT/CT), which were useful to evaluate treatment response to preoperative chemotherapy. Pretreatment radiograph images of the lower leg showed cortical thickening eroded by a broad-based soft-tissue mass without the involvement of the underlying cortex, while computed tomography (CT) revealed a small juxtacortical mass with thick calcification and periosteal reaction. In magnetic resonance imaging (MRI), the mass showed hypointensity in the inner part and isointensity in the outer part in T1-weighted images, while the inner part showed hypointensity and the outer part hyperintensity in T2-weighted images. Bone SPECT/CT results indicated the focal and intense uptake of the mass. Following neoadjuvant chemotherapy (NAC), radiograph and MRI results revealed a slight increase in size, with growing calcification. Although visual inspection of the bone SPECT/CT findings showed nearly the same amount of focal uptake, quantitative parameters determined with those findings were decreased, with maximum standardized uptake value (SUV), peak SUV, mean SUV, metabolic bone volume (MBV), and total bone uptake (TBU) reduced by -20.7%, -22.0%, -12.6%, -33.5%, and -41.9%, respectively. The excision biopsy at the surgery showed a pathological grade 1 (non-complete response) after NAC, including a more than 20% of cell necrosis part. The quantitative bone SPECT/CT was considered to reflect treatment response in this case.

## Introduction

A periosteal osteosarcoma is an extremely rare osteoid-producing sarcoma that arises on a bone surface. Pathological results show this to be an intermediate to a high-grade tumor, thought to arise from the inner layer of the periosteum. Furthermore, histologic assessment reveals largely chondroblastic tissue with smaller areas of osteoid formation. Similar to high-grade intramedullary (conventional) osteosarcomas, these lesions are generally detected in young patients (second or third decade of life) and most frequently (85 - 95%) involve the femur and tibia, followed by the ulna and humerus (5 - 10%) [1-2]. Since a periosteal osteosarcoma is a locally aggressive malignant tumor, an inadequate surgical resection can result in recurrence; thus, the currently recommended treatment consists of both surgical resection and neoadjuvant chemotherapy (NAC).

Bone scintigraphy with technetium 99m (^99m^Tc) examinations are widely used to evaluate osteoblastic activity. However, it is technically difficult to quantify local tracer uptake using conventional bone scintigraphy, even with images acquired using single photon emission computed tomography (SPECT). On the other hand, recent advances in the integration of computed tomography (CT) for attenuation correction, together with a sophisticated reconstruction technique, have enabled the ability to make quantitative measurements with SPECT/CT suitable for derivation of the standardized uptake value (SUV) [3-4]. It is considered that quantitative SPECT/CT may soon have an enormous clinical effect as an imaging biomarker in the practice of modern nuclear medicine. Here, we report a case of periosteal sarcoma in which quantitative values determined with bone SPECT/CT measurements were useful to evaluate treatment response to NAC.

## Case presentation

**Figure 1:**
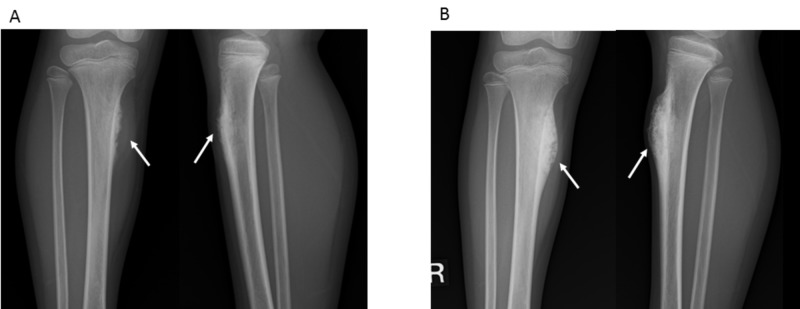
Radiograph of tibial periosteal osteosarcoma before and after neoadjuvant chemotherapy (NAC) A) Pre-neoadjuvant chemotherapy (NAC) anteroposterior and lateral radiograph of the lower leg show cortical thickening that is eroded by a broad-based soft tissue mass without involvement of the underlying cortex; B) Post-NAC anteroposterior and lateral radiograph of the lower leg show cortical thickening that is eroded by a broad-based soft tissue mass with intense calcification.

**Figure 2:**
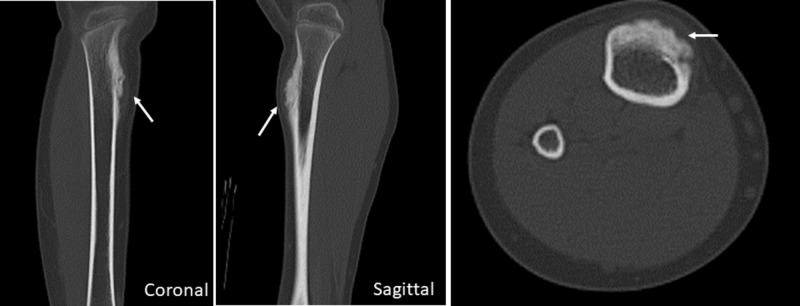
Computed tomography (CT) of the tibial periosteal osteosarcoma before neoadjuvant chemotherapy (NAC) Pre-neoadjuvant chemotherapy (NAC) non-enhanced coronal and sagittal two-dimensional reconstruction images and axial image with bone setting of the lower leg show a juxtacortical mass with thick calcification and periosteal reaction.

**Figure 3:**
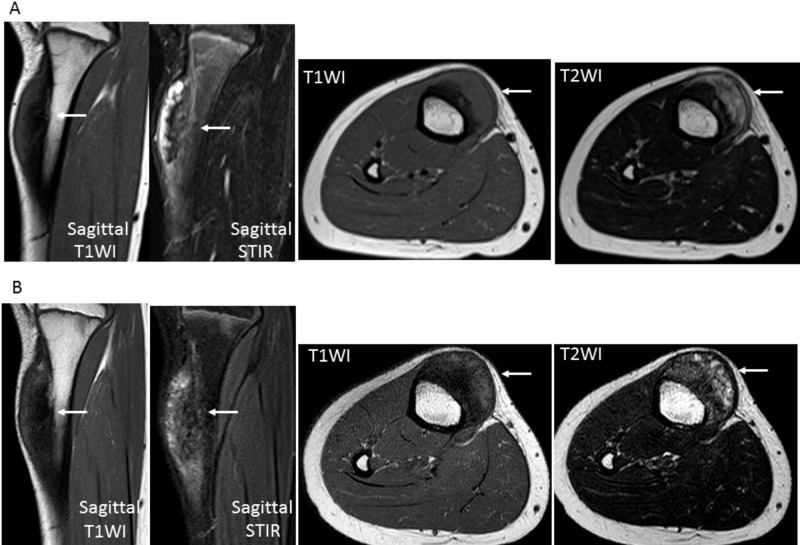
Magnetic resonance imaging (MRI) of the tibial periosteal osteosarcoma before and after neoadjuvant chemotherapy (NAC) A) Pre-NAC MRI shows the mass as a hypointense inner part and isointense outer part on axial and sagittal T1-weighted images and hypointense inner part and hyperintense outer part on axial T2-weighted images and sagittal STIR; B) Post-NAC MRI shows the mass as a growing hypointense inner part and isointense outer part on axial and sagittal T1-weighted images and growing a mixed hypo- and slight hyperintense inner part and hyperintense outer part on axial T2-weighted images and sagittal STIR. STIR: short T1 inversion recovery; T1WI: T1-weighted images; T2WI: T2-weighted images

**Figure 4:**
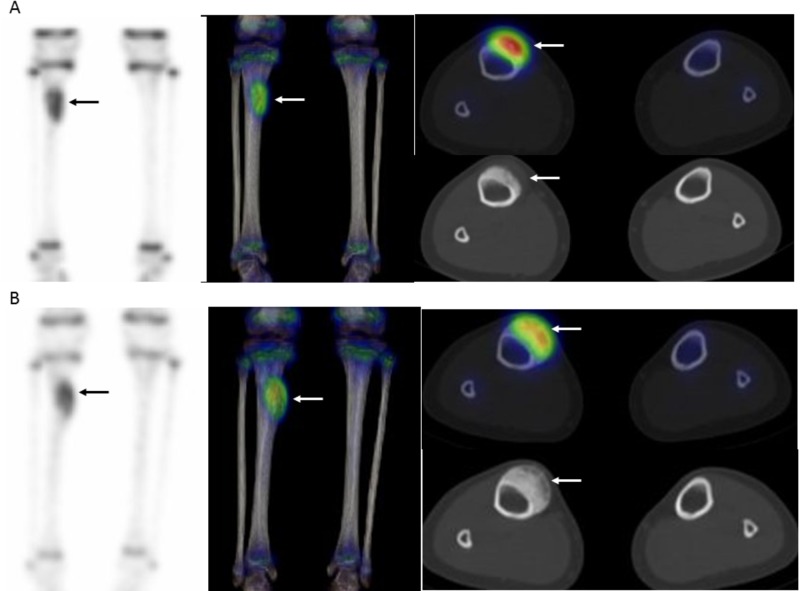
99mTc HMDP SPECT/CT of the tibial periosteal oseteosarcoma before and after neoadjuvant chemotherapy (NAC) A) Pre-NAC bone single photon emission computed tomography/computed tomography (SPECT/CT) (maximum intensity projection (MIP), coronal, and axial SPECT/CT) shows a focal and intense uptake of the mass; B) Post-NAC bone SPECT/CT (MIP, coronal, and axial SPECT/CT) shows the focal uptake with almost no change. ^99m^Tc HMDP: technetium 99m hydroxymethylene diphosphonate

A 10-year-old female came to us with painful swelling in the medial portion of the right lower leg. Her past medical history included asthma, while hematologic and biochemical findings were normal. Radiography of the lower leg showed cortical thickening eroded by a broad-based soft tissue mass without the involvement of the underlying cortex (Figure 1A). In CT findings, a small juxtacortical mass with thick calcification was seen, along with a periosteal reaction on the surface of the right tibia (Figure 2). Magnetic resonance imaging (MRI) revealed a mass with a hypointense inner segment and an isointense outer segment in the axial and sagittal T1-weighted images and a hypointense inner segment and a hyperintense outer segment in the axial T2-weighted images, as well as sagittal short T1 inversion recovery (STIR) (Figure 3A). Technetium 99m (^99m^Tc) hydroxymethylene diphosphonate (HMDP) bone SPECT/CT findings showed a focal and intense uptake by the mass (Figure 4A). Based on the radiological results, the differential diagnosis included a primary surface bone tumor, such as periosteal osteosarcoma, conventional chondroblastic osteosarcoma, and chondrosarcoma, as well as a soft tissue tumor with secondary marrow invasion. An incisional biopsy specimen was obtained from the mass, which demonstrated a malignant tumor with chondrosarcomatous features. The pathological diagnosis was periosteal osteosarcoma. Two courses of NAC with methotrexate, adriamycin, and cisplatin were administered.

**Figure 5:**
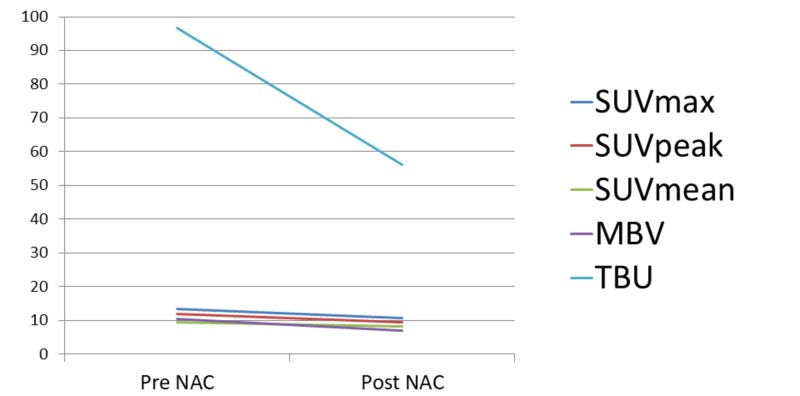
Maximum standardized uptake value (SUV), peak SUV, mean SUV, metabolic bone volume (MBV), and total bone uptake (TBU) of the primary tumor before and after neoadjuvant chemotherapy (NAC) The maximum standardized uptake value (SUVmax), SUVpeak, SUVmean, metabolic bone volume (MBV), and total bone uptake (TBU) of the untreated primary tumor were 13.45, 12.03, 9.32, 10.36, and 96.57, respectively. After neoadjuvant chemotherapy, those parameters decreased a little to 10.68, 9.38, 8.15, 6.89, and 56.14, respectively. The decreasing change of those parameters were -20.7%, -22.0%, -12.6%, -33.5%, and -41.9%, respectively.

Following NAC, radiography, MRI, and ^99m^Tc HMDP bone SPECT/CT examinations were performed. Radiograph images showed a broad-based soft tissue mass with intense calcification (Figure 1B) and MRI revealed growth of the inner section corresponding to the calcification (Figure 3B), while visual examination of the ^99m^Tc HMDP bone SPECT/CT images showed nearly the same level of focal uptake as compared to before the NAC (Figure 4B). The sizes of the mass before and after NAC were 12 × 29 × 62 mm and 17 × 29 × 62 mm, indicating a mild growth. Next, two SPECT/CT scans were performed using an integrated SPECT/CT system (Discovery™ NM/CT 670) (GE Healthcare, Chicago, IL) equipped with a low-energy, high-resolution collimator three hours after an intravenous injection of 440 MBq (megabecquerel) of ^99m^Tc HMDP. The data obtained were analyzed using a commercially available software package (GI-BONE) (Aze Co., Ltd., Tokyo, Japan), which presents values for various SUVs, including max, peak, and mean SUV, metabolic bone volume (MBV), and total bone uptake (TBU). SUVmax represents the single greatest point of metabolic activity within the tumor. SUVpeak is defined as average activity concentration within a 1 cm^3^ spherical volume of interest (VOI) centered on the “hottest focus” within the tumor. The average value of the SUV, which showed 40% or more of the SUVmax in the VOI, is defined as the SUVmean. MBV is defined as tumor volume with uptake. Total lesion glycolysis (TLG) was calculated as SUVmean × MBV. The SUVmax, SUVpeak, SUVmean, MBV, and TBU values of the mass before NAC were 13.45, 12.03, 9.32, 10.36, and 96.57, respectively, while those after NAC were decreased slightly to 10.68, 9.38, 8.15, 6.89, and 56.14, respectively, for reductions of -20.7%, -22.0%, -12.6%, -33.5%, and -41.9%, respectively (Figure 5).

The patient underwent surgery and intraoperative extracorporeal radiation therapy. After making a wide excision, soft and tumor tissue on the tibial surface were removed, then irradiation was performed with a 50 Gy dose, followed by re-implantation in the original site and fixing with a plate. The excision biopsy at the surgery showed a pathological grade 1 (non-complete response) after NAC, including a more than 20% of cell necrosis section. The quantitative bone SPECT/CT was considered to reflect the treatment response in this case.

## Discussion

It has been reported that the histologic response to preoperative chemotherapy is one of the most important prognostic factors for predicting the survival of patients with a periosteal osteosarcoma [2]. However, tumor necrosis as an indicator of histologic response is typically checked by examination of the resected specimen after surgery, and it is difficult to differentiate histologic responders from non-responders to NAC prior to surgery. Information regarding histologic response obtained prior to surgical resection would be helpful for making treatment decisions in advance. Therefore, non-invasive imaging methods, such as CT, MRI, and fluorodeoxyglucose (FDG)-PET/CT, have been studied extensively for use in checking histologic response [5], whereas this is the first report of the use of quantitative bone SPECT/CT.

State-of-the-art SPECT/CT produces objective quantitative data. Using results of robust algorithms of CT-based attenuation correction, scatter correction, and resolution recovery, SPECT/CT is able to generate imaging voxels denoted as units of radioactivity per volume (i.e., kilobecquerels (kBq)/ml). This is fundamentally different from traditional nuclear imaging methods, such as planar scintigraphy, SPECT, or nonquantitative SPECT/CT, which use counts per second for their imaging units. Using quantitative SPECT/CT, lesion radioactivity can be normalized for the injected radioactivity, resulting in quantitative parameter values, such as percent injected dose and SUV [3-4]. Zeintl et al. [3] reported that advanced SPECT/CT technology facilitated quantitative ^99m^Tc SPECT imaging with excellent accuracy in both phantom (error < 3.6%) and patient (error < 1.1%) studies. Furthermore, Gnesin et al. [6] showed that the absolute activity and concentration of activity determined with quantitative ^99m^Tc SPECT/CT were within 10% of the expected values in a phantom study.

Although several recently published studies have demonstrated the clinical application of quantitative SPECT/CT for bone and joint imaging [7-10], there are no known reports of the use of quantitative SPECT/CT for osteosarcoma patients. Suh et al. [7] observed that SUVmax in quantitative bone SPECT/CT measurements gradually increased from normal (2.82 ± 0.73) to mild or moderately abnormal (3.56 ± 0.76, p < 0.05) and then to severely abnormal (4.86 ± 1.25, p < 0.05) in patients with a temporomandibular joint disorder. They concluded that SUVmax based on quantitative bone SPECT/CT may be useful for evaluation of temporomandibular joint disorder. Yamane et al. [9] also observed that the SUVmax value determined from quantitative bone SPECT/CT findings was increased in the epiphyseal plates of children under the age of 15 years in comparison with an older group (25.7 ± 3.2 vs 8.1 ± 4.1, p < 0.0001), which corresponded to higher levels of osteoblastic activity, and concluded that the ability to determine SUV values based on quantitative bone SPECT/CT results has the potential to improve the management of children with a growth disorder.

Lee et al. [11] compared ^99m^Tc methyl diphosphonate (MDP) bone scintigraphy and FDG-PET/CT for predicting histologic response to NAC in 62 patients with osteosarcoma. They demonstrated that the sensitivity and specificity for predicting good histologic response were 83.3% and 75%, for the criterion the percent change of the maximum tumor-to-nontumor ratio < -12.5% on ^99m^Tc-HMDP bone scintigraphy, and 80.0% and 81.3% for the criterion the percent change of SUVmax < -49.0% on FDG-PET/CT, respectively. Because planar bone scintigraphy was scanned, SUV could not be calculated. Wakabayashi et al. [12] compared ^99m^Tc-hexakis2-methoxyisobutylisonitrile (MIBI) scintigraphy and contrast-enhanced MRI for predicting histologic response to NAC in 17 patients with osteosarcoma. They demonstrated that the sensitivity, specificity, and accuracy for predicting good histologic response was 100%, 75%, and 88%, respectively, on ^99m^Tc-MIBI scintigraphy, and 88%, 100%, and 94%, respectively, on contrast-enhanced MRI. Palmerini et al. [13] evaluated the diagnostic performance of FDG-PET/CT for predicting histologic response to NAC and recurrence in 45 patients with Ewing sarcoma and 32 patients with osteosarcoma. They demonstrated that a good metabolic response (SUV reduction of ≥ 55%) was associated with three-year event-free survival of 80% and a poor metabolic response with a three-year event-free survival of 20% (p = 0.05).

Radiography, CT, and MRI findings have characteristic features [1, 14]. The radiologic appearance of a periosteal osteosarcoma demonstrates a broad-based surface soft-tissue mass causing extrinsic erosion of the thickened underlying diaphyseal cortex and a perpendicular periosteal reaction extending into the soft tissue component. CT provides other valuable information regarding the tumor origin and matrix, calcification within the soft tissue mass, and mass localization. Differentiation from more common lesions may be difficult using radiography. CT is useful for differentiation in cases of chondroid tumor, in which a hypodense chondroid matrix and punctate calcification are specific findings. Furthermore, MRI may more precisely reveal the margin and extent of the mass and can also provide information for a differential diagnosis. A periosteal osteosarcoma shows a similar level of signal intensity as muscle in T1-weighed images, while a heterogeneous high-signal intensity is seen in T2-weighted images, which reflects the predominance of the chondroid components within the mass. Additionally, peripheral or septal patterns of contrast enhancement also suggest a cartilaginous lesion, while adjacent bone may show focal areas with a signal change due to reactive marrow edema. Less commonly, intramedullary invasion by the tumor may be seen as continuity from the soft tissue mass. Such MR findings are helpful for differentiating a periosteal osteosarcoma from a high-grade surface osteosarcoma, which does not show high-signal intensity in T2-weighted images or peripheral enhancement due to the lack of high-water content and a cartilaginous component. On the other hand, MR imaging is less sensitive than CT for the detection of mineralization in the mass.

## Conclusions

Various indexes, such as SUVmax, SUVpeak, SUVmean, MBV, and TBU determined using quantitative bone SPECT/CT findings, may be useful to evaluate the activity of an osteosarcoma. Additional studies with a larger cohort are needed in order to determine the clinical impact of quantitative bone SPECT/CT on the evaluation of treatment response in patients with bony lesions.
